# Label-free Surface Enhanced Raman Scattering (SERS) on Centrifugal Silver Plasmonic Paper (CSPP): A Novel Methodology for Unprocessed Biofluids Sampling and Analysis

**DOI:** 10.3390/bios11110467

**Published:** 2021-11-21

**Authors:** Alessandro Esposito, Alois Bonifacio, Valter Sergo, Stefano Fornasaro

**Affiliations:** Raman Spectroscopy Lab, Department of Engineering and Architecture, University of Trieste, 34100 Trieste, Italy; ALESSANDRO.ESPOSITO@phd.units.it (A.E.); abonifacio@units.it (A.B.); sergo@units.it (V.S.)

**Keywords:** label-free SERS, biofluids, plasmonic paper, vibrational spectroscopy, serum

## Abstract

Label-free SERS is a powerful bio-analytical technique in which molecular fingerprinting is combined with localized surface plasmons (LSPs) on metal surfaces to achieve high sensitivity. Silver and gold colloids are among the most common nanostructured substrates used in SERS, but since protein-rich samples such as serum or plasma can hinder the SERS effect due to protein–substrate interactions, they often require a deproteinization step. Moreover, SERS methods based on metal colloids often suffer from a poor reproducibility. Here, we propose a paper-based SERS sampling method in which unprocessed human serum samples are first soaked on paper strips (0.4 × 2 cm^2^), and then mixed with colloidal silver nanoparticles by centrifugation to obtain a Centrifugal Silver Plasmonic Paper (CSPP). The CSPP methodology has the potential to become a promising tool in bioanalytical SERS applications: it uses common colloidal substrates but without the need for sample deproteinization, while having a good reproducibility both in terms of overall spectral shape (r > 0.96) and absolute intensity (RSD < 10%). Moreover, this methodology allows SERS analysis more than one month after serum collection on the paper strip, facilitating storage and handling of clinical samples (including shipping from clinical sites to labs).

## 1. Introduction

Surface enhanced Raman scattering (SERS) spectroscopy is a powerful analytical technique that combines easy and rapid data acquisition with high sensitivity and specificity in chemical species recognition. The capability in Raman spectroscopy of identifying molecular species is usually referred to as vibrational fingerprinting and is obtained by recording specific vibrational modes of sampled molecules. In SERS, metal nanostructured surfaces with proper plasmonic features (i.e., the SERS substrates) can enhance by several orders of magnitude the Raman signal intensity of adsorbing molecules, by hot-spots formation in which LSPRs (localized surface plasmon resonances) are generated [1,2]. 

SERS substrates can be roughly divided into solid and colloidal substrates. The most common colloidal substrates are produced via a chemical synthesis of colloidal dispersions of gold and silver nanoparticles (NPs) in aqueous media [3,4]. In these substrates, a strong LSPR interaction is obtained by the aggregation of NPs in the presence of analytes. Alternatively, depositing pre-aggregated or aggregating nanoparticles on different types of solid supports, such as porous silicon or paper [5,6], is a common approach to produce solid or flexible substrates, respectively. These substrates allow for a more stable and controlled distribution of metal nanoparticles that interact with adsorbing molecules. In contrast, it is arduous to adequately control the NP aggregation of the colloid–sample mixture in colloidal systems, resulting in high level of SERS signal variability due to the presence of a different hot-spot concentration and large aggregate precipitation [7].

In particular, paper-based SERS substrates, also known as plasmonic papers, have gained importance in both the number of applications and production techniques, thanks to their generally inexpensive and easy production procedures [8]. In this context, nanoparticles in situ growth and post-immobilization are the two more common methods to produce plasmonic papers. In the latter method, nanoparticles are produced using the cellulose matrix as a 3D scaffold, and its lateral chains as reducing agents for silver and gold salts [9]. The post immobilization process can be achieved by different methods: dip-coating [10,11], filtration [12], printing [13], and vapor deposition [14]. It is therefore possible to adapt the fabrication process for specific analytical needs [15], but with two main limitations: reproducibility and a relatively low throughput of fabrication. Aside from reproducibility, which is a common issue in SERS substrates [16], a more general drawback affecting all paper SERS applications is production throughput. This aspect is an important limiting factor in the commercialization [17], considering that each protocol can be applied for one of a few applications. These factors have limited a real commercial application of plasmonic papers in the SERS analytical field, although they remain a viable tool at the laboratory scale [17,18]. For instance, paper-based substrates appear as a perfect choice for the label-free SERS analytical “vocation” that aims to produce a point-of-care technique, with compact and portable instrumentation, allowing fast, user-friendly, and cheap measurements. However, very few paper-based substrates have been applied in the label-free SERS analysis of unprocessed biofluids [19,20], in which the complexity of the biological samples can be a non-trivial aspect to be tackled, in terms of the chemical–physical environment and the presence of possible disturbing factors at the nano-bio interface [21]. 

One of the main challenges of using noncolloidal substrates for label-free SERS analysis of biofluids is an unspecific binding of proteins onto the sensing interface of substrates (i.e., the so-called “protein fouling”). Pre-treatment methods such as protein precipitation, filtration, or separation techniques such as chromatography are commonly employed to tackle this issue before actual SERS sensing, resulting in delayed results and higher costs for analysis [16].

We have previously addressed these issues by sampling a biofluid before the post-immobilization process, resulting in strong and reproducible SERS signals [22]. Here, we extend our previous work by optimizing a novel protocol for label-free SERS sensing of unprocessed human serum. Specifically, the Centrifugal Silver Plasmonic Paper (CSPP) protocol utilizes an innovative approach using common filter paper strips for serum sampling, followed by a post-immobilization process of silver NPs achieved by centrifugation.

## 2. Materials and Methods

### 2.1. Materials and Regents

All chemicals (analytical grade), human serum (from male AB plasma, frozen liquid, USA origin), Whatman filter paper (grade 4), and all other reagents unless stated otherwise were purchased from Merck (Darmstadt, Germany) and used as obtained without further purification. Ultrapure deionized (DI) water of 18.2 MΩ cm resistivity at 25 °C was used throughout the experiments and it was obtained by a Millipore Milli-Q system (Merck, Darmstadt, Germany). Sulfuric and nitric acid for glassware cleaning was purchased by VWR (Avantor, Radnor, PA, USA). Ergothioneine (ERG; purity 99.0%) was obtained from Apollo Scientific (Cheshire, UK). 

### 2.2. Silver Nanoparticle Synthesis and Characterization

Prior to the synthesis, all the glassware was cleaned with a sulfuric acid Nochromix^®^ (Godax Laboratories, Bethesda, MD, USA) solution and nitric acid to remove organic and silver metal residues, respectively, and finally was thoroughly rinsed with MilliQ water. The aqueous colloidal dispersion of silver nanoparticles (Ag-NPs) was synthesized following the Lee–Meisel protocol [4]. Briefly, 45 mg of AgNO_3_ was added to 250 mL of MilliQ water in a flask and heated until boiling. The chemical reduction was achieved by adding 5 mL of sodium citrate tribasic dihydrate solution (1%, g/mL) dropwise in water while keeping boiling for 1 h under vigorous stirring and water condenser reflux. Ultimately, the Ag-NPs dispersion was left to cool down at room temperature and conserved in the dark until use. Ag-NPs were characterized by UV-visible spectroscopy (Cary100, Agilent, Santa Clara, CA, USA) and transmission electron microscopy (TEM, Philips EM 208 Philips Scientifics, Eindhoven, The Netherlands).

### 2.3. Serum Processing

To obtain the processed serum samples, serum aliquots (500 μL) were filtered (12,100 g for 30 min) using an Amicon centrifugal filter unit with a cut-off of 3 kDa (Millipore-Merck, Darmstadt, Germany), pre-rinsed with DI water, as reported in [23].

### 2.4. CSPP Sampling Methodology 

**Figure 1:**
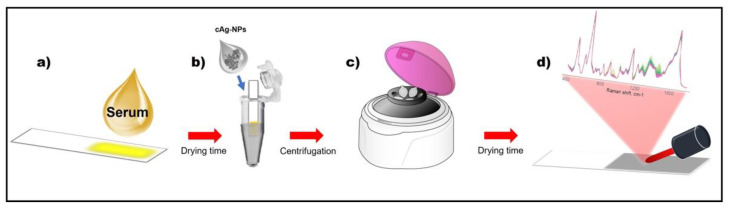
Schematic workflow for CSPP sensing of serum samples.

Figure 1 shows the schematic workflow of the CSPP protocol. Briefly, 2 μL of serum sample was placed into the reversed lid of an Eppendorf tube, used as a reservoir. High quality filter paper (pore size = 2 µm) was cut into strips (0.4 × 2 cm^2^) using normal scissors. The paper strips were vertically placed into the lids, ensuring that the strip came into contact with the liquid. The serum samples were allowed to soak though the paper short edge for 30 s. The serum-soaked strips were then removed from the lid and left to dry. Dried strips were then put into separate sealed Eppendorf tubes containing 75 μL of Ag-NPs, with the serum-soaked part pointing toward the bottom of the Eppendorf, and then centrifuged to obtain a CSPP. After the centrifugation, the CSPP was left to dry prior to SERS analysis. The morphology of the CSPPs was identified using scanning electron microscopy (SEM) analysis using an FESEM-FIB Olympus (Zeiss, Oberkochen, Germany).

### 2.5. SERS Spectral Acquisition and Data Processing

SERS spectra were collected at room temperature (22 ± 0.5 °C) with a portable i-Raman plus spectrometer (B&W Tek, Plainsboro, NJ, USA) equipped with a 785 nm laser (output 400 mW) and connected with a compact microscope mounting an Olympus optics 20X (N.A. 0.25, working distance 8.8 mm) with a spot size of 108 µm. For data collection, the laser power was reduced to 5% (18 mW) and an exposure time of 10 s was used to avoid sample photodegradation. Spectra from 10 different spots were randomly collected for each CSPP (from the grey region, indicating the presence of Ag-NP aggregates), and averaged to accommodate intra-substrate variability. The spectral acquisition was performed using the BWSpec™ version 4.03_23_c (B&W Tek., Newark, DE, USA) software in the Raman shift range of 62–3202 cm^−1^. The BWSpec™ software allowed for the collection of a background signal (dark) before data acquisition and its subtraction from the collected data. Wavenumber calibration was checked before and during every spectral acquisition session by collecting a spectrum of paracetamol as a standard reference. Spectra were imported and processed within the R software environment (version 4.0.1 – “See Things Now”) for statistical computing and graphics, building on the *hyperSpec* [24] and *ggplot2* [25] packages. Spectra were cropped, smoothed, and interpolated using the function *spc.loess* (n = 2.5, spectral range = 400–1800 cm^−1^) from the *hyperSpec* package. Baseline correction was obtained combining a fifth-order polynomial baseline with a classic rubber-band correction (*spc.fit.poly.below* and *rubberband* functions with poly = 5, noise = 100, df = 10). 

### 2.6. Experimental Design 

To optimize the CSPP sampling protocol, six factors at two or three levels related to time and reagent amounts were investigated (Table 1). A reduced second-degree polynomial model having 16 coefficients was proposed (1 constant, 6 linear terms, 6 interactions, and 3 quadratic terms). Interaction terms were introduced for the factor for which an interaction was expected; quadratic terms were considered for the factors for which an optimum condition was expected. In this case, 216 candidate experiments were defined by listing all possible combinations of the considered factors. A D-Optimal design was applied to select the minimum number of experiments to build an informative model for studying the effects of factors and their interactions. The selected runs were chosen by Fedorov’s exchange algorithm, among the 216 candidate experiments, so as to meet the D-Optimality criterion, which consists of the maximization of the determinant of the information matrix (X’X). Four extra runs close to the center of the data space were manually added to validate the model and the total number of runs was determined to be 25 (Table S1). The average integrated areas under the well-defined uric acid SERS band (620–660 cm^−1^), and cellulose normal Raman band (1020–1100 cm^−1^) were calculated, and their ratio was used as a measure of the formation of the CSPP, the response to be maximized (Y). The experimental matrices and models were computed using packages *AlgDesign* [26] and *rsm* [27], respectively. 

### 2.7. Robustness and Experimental Variability

The reproducibility of the results was assessed considering 12 replicated CSPP runs (10 spectra for each CSPPs) at the optimal conditions, and expressed as relative standard deviation (RSD, %) and Pearson correlation coefficient (r). Pearson correlation was chosen as a metric for SERS spectral similarity as, unlike RSD, it does not assume that the level of noise is proportional to the level of enhancement, as proposed by Jarvis et al [28]. 

### 2.8. Serum Collecting System Shelf Life

To evaluate the storage stability in time of the serum-soaked strips, the shelf life was assessed up to two months. Measurements were performed at 4, 16, 24, 216, 384, 720, and 1000 h after serum sampling, with three strips at each time tested. Prior to the SERS analysis, the serum-soaked strips were centrifuged with the Ag-NPs colloidal solution, and the CSPP were obtained at the optimal experimental conditions.

### 2.9. Sensibility

To test the sensibility of the CSPP method to spot differences in the concentration of selected metabolites, human serum was spiked with known amounts of ergothioneine (ERG) and hypoxanthine (HX). (i) First, 4 μL of a 5 mM ERG stock solution prepared in DI was added to 396 μL of serum to obtain a 50 μM serum ERG-spiked stock solution. Amounts of 25, 10, and 5 μM solutions were obtained by 2-fold, 5-fold, and 10-fold dilution in serum. (ii) Next, a 10 mM HX stock solution was prepared in DI water from a 50 mM mother solution in 0.25 mM NaOH, by a 5-fold dilution. A total of 4 μL of the HX stock solution was added to 396 μL of serum to obtain the 100 μM HX-spiked working solution. Finally, 50, 25 and 10 μM solutions were obtained by further 2-fold, 4-fold, and 10-fold dilution in serum, respectively.

## 3. Results and Discussion

### 3.1. Development of the Analytical Method

Filter paper is not only an easy alternative way to collect biological material; it is also a promising sampling support to simplify and strengthen the label-free analysis of biofluids with SERS. Recently, we demonstrated that the use of a very specific commercial paper (Periopaper^®^) coupled with a centrifugal-driven aggregation of Ag-NPs, allowed for a rapid and reproducible analysis of gingival crevicular fluid with SERS without the need for a deproteinization step [22]. The first objective of the present work was to investigate whether the same strategy could be applied for the analysis of a protein-rich biofluid such as unprocessed human serum. A standard commercial serum sample was employed for the method development to ensure sample homogeneity and remove irreproducibility due to individual donors and serum preparation. The feasibility of the CSPP approach proposed here was then investigated by performing experiments both in processed and unprocessed serum samples. In the case of processed samples, molecules smaller than a 3 kD cut-off value diffuse through the pores of the filter unit with an exerted centrifugal force, while larger molecules remain in the filter unit.

**Figure 2:**
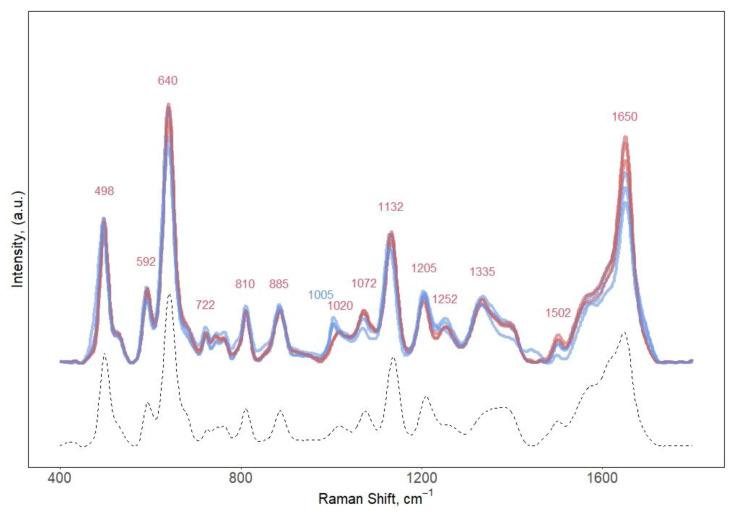
Comparison between the SERS spectra obtained with CSPPs of unprocessed (blue) and filtered (red) human serum (n = 3). SERS spectrum of uric acid (125 μM, dissolved in water) is reported as reference (dashed line). Excitation wavelength: 785 nm.

Figure 2 shows the 785 nm excited SERS spectrum of unprocessed and filtered human serum obtained with the CSPP protocol. We can observe that the most easily recognizable characteristic features in the SERS spectra are practically the same for both sets of samples, as no interference from proteins was detected. Considering the high number of proteins (6–8 g/dL) normally present in serum [29], it is remarkable that the only (small) visible difference in the unfiltered serum spectra is the band centered at 1005 cm^−1^, associated with aminoacidic aromatic residues (Phe ring breathing). On the contrary, both sets of SERS spectra were dominated by the vibrational modes associated with uric acid (UA, bands centered at 640, 812, 885, 1132, 1205, and 1650 cm^−1^) [30]. Interestingly, spectral features of hypoxanthine (HX) and ergothioneine (ERG), often reported in literature as prominent contributors in SERS spectra of biofluids [23,31,32], were missing. A possible explanation can reside in the origin of the serum used in our study. The variation in relative intensity in HX and ERG bands has been linked to the spectral variability due to different clinical states of patients [33,34,35]. With respect to that, a standard commercial serum can easily present a lower HX and ERG concentration since no metabolite standardization or quantification is available for commercial serum.

### 3.2. Characterization of the CSPP

**Figure 3:**
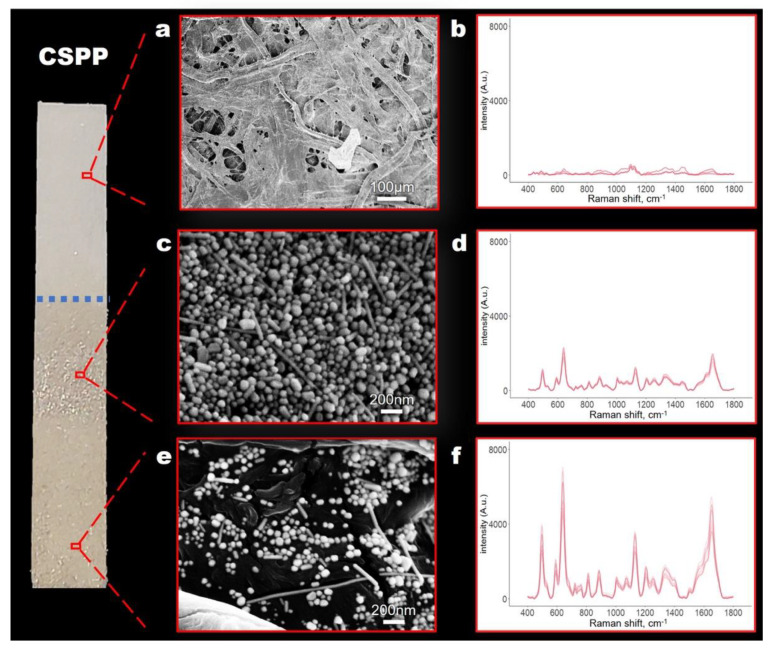
CSPP characterization. Scanning electron microscopy micrographs of different areas on a CSPP (**a**,**c**,**d**) and corresponding SERS spectra (**b**,**d**,**f**). See text for details.

Figure 3 shows the surface morphology of a CSPP, where a non-uniform three-dimensional network can be observed. Three areas with different morphologies related to a different distribution of nanoparticles can be found. On the top of the strip (Figure 3a), no significant differences were observed when compared with raw filter paper. A non-uniform coating of aggregates of spherical Ag-NPs can be observed instead throughout the lower areas of the CSPP (Figure 3c,e), where serum had been previously soaked. This is very important, since it is within these regions that hot spots are created, leading to an increase of the SERS signal. The nonhomogeneous distribution of the aggregates within the CSPP identified two regions with different SERS enhancement due to variation in SERS hot-spots formation. This feature can easily be associated with the centrifugation process using a fixed-angle rotor: NPs sink though the 3D structure of the cellulose fibers during centrifugation until a certain position where they locally concentrate, driven by centripetal force. During the centrifugation process the paper strip bends, forming a U-shaped structure against the walls of the centrifugation tube. This gravity-induced deformation is probably the cause of the inhomogeneous NP distribution on the paper surface. Interestingly, the area in the middle of a CSPP, with a higher concentration of nanoparticles (Figure 3c), exhibited a reduced SERS enhancement (Figure 3d) compared to the area at the tip (Figure 3e,f). A possible explanation could be that a lesser fraction of the analyte molecules is able to reach the hot spots on the top NP layers by diffusing from the paper below through the thick NPs deposit. Most of the analyte probably remains adsorbed on the NPs in deeper layers, shielded by the upper layers, and thus virtually inaccessible to the exciting laser. 

On the other hand, we are unable to offer suggestions about the modalities of the serum distribution along the strip. A simple colorimetric test (i.e., blue Coomassie paper staining) was performed to spot the protein distribution, but no specific pattern was observed (data not shown), suggesting a homogeneous distribution.

### 3.3. Study of the Impact of Independent Factors on the SERS Signal

The second objective was to optimize the CSPP protocol for the SERS analysis of human serum. The experimental matrix, made by 21 experiments total, allowed the coefficients of the model to be estimated with optimal quality (the highest inflation factor was 1.77) and with 5 degrees of freedom. The corresponding values of the factors range-scaled between −1 and + 1, and the values of response variable Y, are collected in Table S1. Y (the ratio between the areas under the SERS band of uric acid, centered at 640 cm^−1^ and cellulose Raman band, centered at 1040 cm^−1^) varies in response to the success of the post-immobilization process. Therefore, higher values of Y correspond to a decrease in background contribution derived from the paper strip. The relationship between the response variable Y and the factors (as coded values) is explained by the model reported in Equation (1), in which the factors are coded according to Table 1.

Y = 4.97 − 0.61A − 0.37B + 0.80C + 1.82D (**) − 0.54E + 2.01F (**) + 0.83BC + 0.30 BD + 0.10BE + 0.43CD + 1.03CE − 0.84 DE + 3.66 B^2^ (*) + 0.29 D^2^ − 0.72 E^2^ (1)

**Figure 4:**
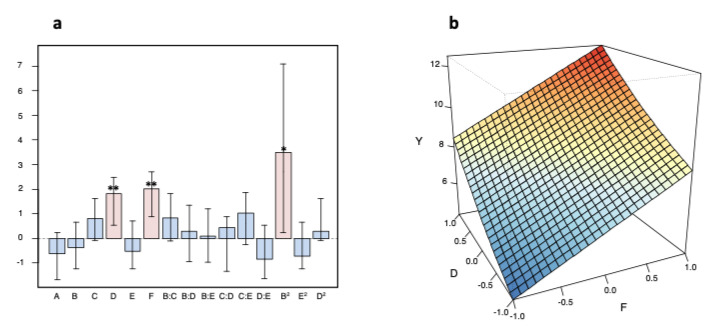
(**a**) Plot of the coefficients (whiskers indicate the confidence interval at *p* = 0.05; * = *p* < 0.05, ** = *p* < 0.01; the *y*-axis (dimensionless) shows the magnitude of the coefficients, which is identical to the coefficient values reported in Equation (1). Experimental factors are coded as reported in Table 1. B:C, B:D, B:E, C:D, C:E, and D:E, are the two-term interactions. (**b**) Response (Y) predicted by the experimental design model as a function of D (centrifugation time) and F (drying time after centrifugation), while keeping A = 0 (10 min), B = 1 (150 “L), C = 0 (10 min), and E = 0 (8000 rpm).

Asterisks in this equation indicate the significance of the coefficients (* = *p* < 0.05; ** = *p* < 0.01). The model explains 73.8% of the variance in fitting, with a standard deviation of the residuals of 1.7. The experimental values obtained at the four validation points were not significantly different from the predicted values (Table S2), therefore validating the model results. Figure 4a shows the plot of the model coefficients. The significant coefficients are the linear terms for the factors D (centrifugation time) and F (drying time after centrifugation) plus the quadratic term for B (Ag-NPs volume). This means that an increase of D and F causes a significant increase in the response Y, SERS spectral intensity. Three factors (analyte initial drying time, A; incubation time, C; and centrifugation speed, E) and all interactions between pairs of factors (BC, BD, BE, CD, CE, DE) had non-significant coefficients and therefore can be considered as non-relevant. Regarding B (Ag-NPs volume), a direct interpretation of its effect from the convex quadratic term (B^2^), with a non-significant linear coefficient, is not possible. To obtain a better understanding of the phenomenon, response surfaces must be drawn. Figure 4b shows the response surface on the plane D vs. F, obtained by setting A = 0 (i.e., 10 min), B = 1 (i.e., 150 μL), C = 0 (i.e., 10 min), and E = 0 (8000 rpm). It can be seen that, as expected, an increase of both times produced an increase of the response, and for both factors the effect was larger when the other factor was at a low level. Moreover, the surfaces in Figure S4a,b clearly show how a higher spectral intensity can be obtained only by working at the extremes of the volume range for factor C. The condition corresponding to 20 min of centrifugation and drying time and 75 μL was detected as the optimal condition. 

### 3.4. Precision and Experimental Variability

**Figure 5:**
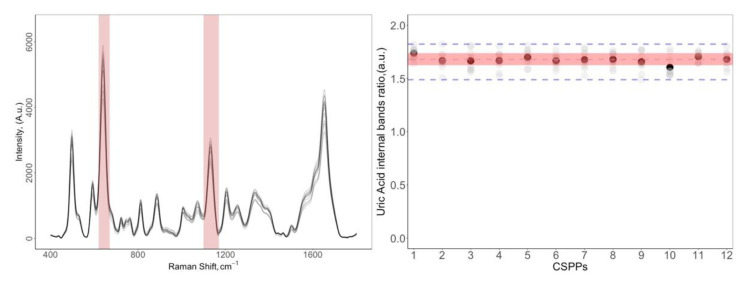
Reproducibility of the CSPP method. (**a**) SERS spectra of 12 different CSPPs; (**b**) Distribution of the ratio between the 640 (skeletal ring deformation) and 1132 (C-N) cm^−1^ bands of uric acid over the 12 CSPPs. Black dots correspond to the mean value of 10 randomly chosen positions on the same CSPP. The limits of the shaded area are the upper and lower quartiles, calculated over the entire set of measurements, so the area spans the interquartile range (IQR), corresponding to the range covered by 50% of the data. The dashed lines mark the median and the extremes of the acceptance area (1.5 × IQR).

An important critical issue for the proposed protocol to be used as a routine analytical tool is the reproducibility. The experimental variability of the CSPPs had to be assessed at the optimal conditions. We evaluated the reproducibility with consecutive measurements from 12 CSPPs prepared at the same time from the same sample using the same measurement procedure, operator, measuring system, and operating conditions. As shown in Figure 5a, the spectra were quite reproducible in terms of ratio between bands (i.e., the overall spectral shape), and did not show any systematic intensity variation that could be associated with the CSPP protocol. The correlation coefficients (r) between spectra ranged between 0.96 and 0.99, showing an excellent level of similarity, with a good level of homoscedasticity.

On the other hand, the relative standard deviation (%RSD), indicated by the absolute intensity variation of the 640 cm^−1^ band computed on 12 CSPPs, was 9.08%. It is evident that with this value of RSD, the method cannot be directly used for quantitative determinations, but it is sufficiently good for studies in which the relevant differences among samples are related to the shape of the signal (*fingerprint*) and not to the global signal intensity (e.g., for many classification studies). The %RSD value dropped to 1.56% when the ratio between two bands of uric acid was considered, reaching an inter-sample precision superior to the ones reported for other paper-based SERS substrates [6,11,36,37,38].

### 3.5. Shelf Life

**Figure 6:**
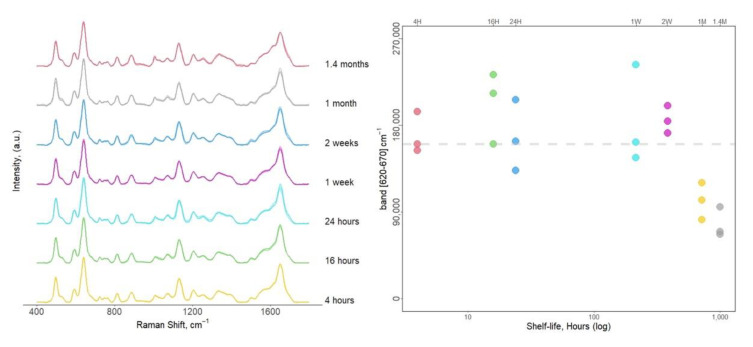
Shelf life evaluation from serum-soaked strips for the CSPP sampling protocol. (**a**) Normalized SERS spectra; (**b**) absolute intensity distribution at 640 cm^−1^ band corresponding to the different times reported in (a). The average intensity of the first time point is marked with a dashed line for comparison.

To thoroughly validate our methodology, the stability of the sample (i.e., a serum-soaked strip) was verified over time. The shelf life of serum-soaked strips was tested for up to two months. Indeed, the analysis of the same sample kept at −20 °C until analysis and analysis on different days did not show any significant difference in the spectral information, even after two months of storage (Figure 6a), with a 50% average decrease of the spectral intensity after one month (Figure 6b). Overall, these results permit a certain degree of freedom in collection and SERS analysis organization. In fact, due to the stability of the signal, larger SERS label-free sampling campaigns are possible.

### 3.6. Evaluation of the SERS Response at Different Metabolite Concentrations

**Figure 7:**
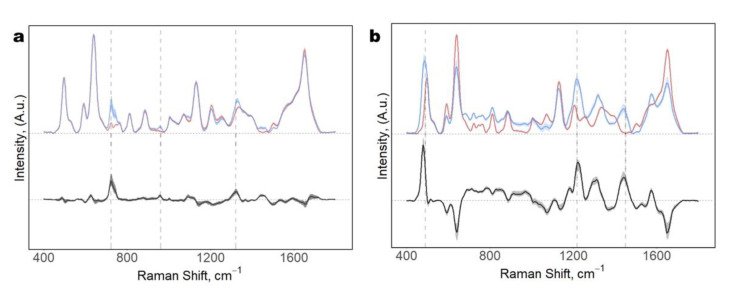
SERS spectra of serum samples spiked with pure metabolites. (**a**) top, over imposed spectra of serum (red) and serum + 50 μM HX (blue); bottom, difference spectrum HX-serum. (**b**) top, over imposed spectra of serum (red) and serum + 25 μM ERG (blue); bottom, difference spectrum ERG-serum.

The high reproducibility of the SERS spectra obtained with CSPPs could imply that the CSPP methodology is insensible to the subtle changes in relative metabolite concentrations, usually observed in the subjects involved in diagnostic or prognostic studies. However, one of the main aims of a reliable bio-analytical method is to discriminate through different relevant biological metabolites and their concentrations. To assess this hypothesis and test the applicability of the proposed methodology, two pure metabolites often observed in the SERS spectra of biofluids, HX and ERG [23,32], were used to spike serum samples in different amounts, to simulate biochemical differences linked to a hypothetical health condition. The spiked samples were analyzed with the CSPP protocol. Intense SERS spectra of these metabolites were observed, showing the main SERS bands of the two probing metabolites, clearly resembling the SERS spectra previously reported in the literature [23,31,32]. Even with tiny variations, such as of 10 μM and 5 μM for the ERG and HX, respectively, we were able to see increments of specific metabolites’ bands, (Figure 7). Moreover, a principal component analysis (PCA) showed a clear separation among the three groups (serum; serum + ERG; serum + HX), indicating the possibility of clearly detecting a metabolite change in concentrations at the micromolar level (Figure S4). 

## 4. Conclusions

In this work, we presented a novel, facile, rapid, and cost-effective method to obtain a label-free SERS spectrum from human serum without the need for a filtration (deproteinization) step. In contrast to conventional methods, in which samples are deposited on already prepared SERS substrates, our method involves a simple sampling step with filter paper followed by a rapid immobilization of citrate-reduced silver nanoparticles as the plasmonic component. Thus, before the collection of the sample, no former paper treatment is necessary. This aspect, together with the high reliability and reproducibility of the obtained spectral information, make the CSPP methodology a promising protocol for SERS of biofluids with the capability to accommodate small volume sample collection by non-medically trained caregivers or by the patients themselves. In this study, the main experimental parameters used in CSPP methodology were systematically examined for optimization: 20 min of centrifugation and drying time and 75 μL of Ag-NPs were detected as the optimal conditions. All the other instrumental variables were demonstrated to be non-relevant in the whole range under study. Moreover, as a proof-of-concept, differences in the concentration of selected serum metabolites were monitored without additional tagging or chemical modification, making the CSPP protocol a good alternative for SERS metabolomic fingerprinting analyses.

## Figures and Tables

**Table 1:** Experimental factors and levels of D-optimal for CSPP optimization.

Factor	Symbol	Levels		
Analyte drying time (min)	A	0	-	20
Ag-NPs volume (µL)	B	75	100	150
Incubation time (min)	C	0	-	20
Centrifugation time (min)	D	2	10	20
Centrifugation speed (rpm)	E	4000	8000	13,000
Drying time after centrifugation (min)	F	0	-	20

## Data Availability

The data presented in this study are openly available in Zenodo at doi:10.5281/zenodo.5644790.

## Supplementary Materials

The following are available online at https://www.mdpi.com/article/10.3390/bios11110467/s1, Figure S1: Serum-soaked paper strip before and after centrifugation; Table S1: Experimental factors and levels of D-optimal design for CSPP optimization; Table S2: Results of model validation; Figure S2: Response surfaces; Figure S3: CSPP reproducibility; Figure S4: Principal components analysis.
