# Supplementary material for: Label-free Surface Enhanced Raman Scattering (SERS) on Centrifugal Silver Plasmonic Paper (CSPP): A Novel Methodology for Unprocessed Biofluids Sampling and Analysis
Source: Biosensors (Basel). 2021 Nov 21;11(11):467. doi: 10.3390/bios11110467 (PMC8615985; doi:10.3390/bios11110467)
Supplement: Supplementary file 1 [file biosensors-11-00467-s001.zip › biosensors-1472516-supplementary.pdf]

biosensors

*Supplementary*

# Label-Free Surface Enhanced Raman Scattering (SERS) on Centrifugal Silver Plasmonic Paper (CSPP): A Novel Methodology for Unprocessed Biofluids Sampling and Analysis

**Alessandro Esposito, Alois Bonifacio, Valter Sergo and Stefano Fornasaro***

Raman Spectroscopy Lab, Department of Engineering and Architecture, University of Trieste, 34100 Trieste, Italy

* Correspondence: sfornasaro@units.it

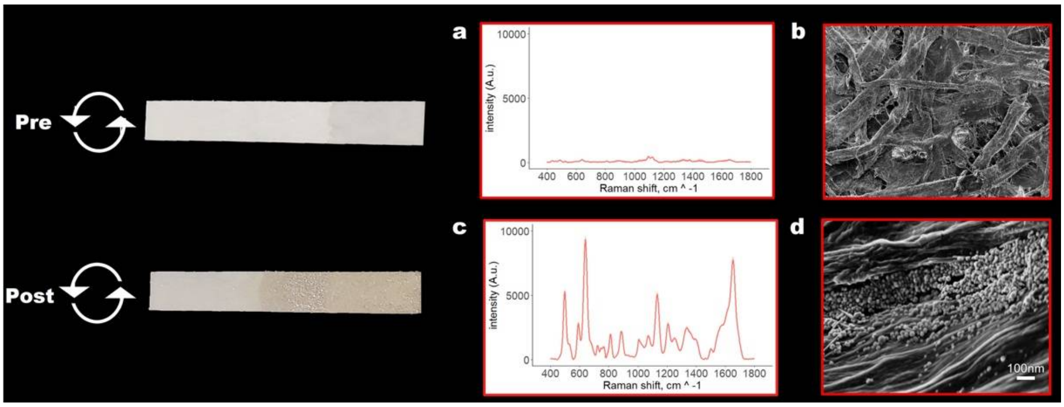


**Figure S1.** Serum-soaked paper strip before and after centrifugation; (a, c), SERS spectra; (b, d) SEM images.

**Table S1.** Experimental matrix reporting the 25 experiments performed, with the sequence of values applied for the six experimental factors and their correspondent range-scaled X variable (A-F), in the D-optimal design for CSPP optimization. M, experimental runs; V, validation runs; Y, response variable (UA/cellulose ratio). tdry, analyte drying time; Vcol, Ag-NPs volume; tinc, incubation time; tcent, centrifugation time; s, centrifugation speed; tdry2, drying time after centrifugation.

| SET | $t_{dry}$ (min) | $V_{col}$ (μL) | $t^{inc}$ (min) | $t_{cent}$ (min) | s (rpm) | $t_{dry2}$ (min) | A (min) | B (μL) | C (min) | D (min) | E (rpm) | F (min) | Y |
|---|---|---|---|---|---|---|---|---|---|---|---|---|---|
| M | 20 | 150 | 0 | 2 | 4000 | 0 | 1 | 1 | -1 | -1 | -1 | -1 | 2.01 |
| | 0 | 75 | 0 | 2 | 13000 | 0 | -1 | -1 | -1 | -1 | 1 | -1 | 5.21 |
| | 20 | 150 | 0 | 20 | 4000 | 0 | 1 | 1 | -1 | 1 | -1 | -1 | 9.11 |
| | 0 | 75 | 20 | 2 | 4000 | 20 | -1 | -1 | 1 | -1 | -1 | 1 | 8.64 |
| | 20 | 150 | 0 | 2 | 13000 | 0 | 1 | 1 | -1 | -1 | 1 | -1 | 0.84 |
| | 0 | 75 | 0 | 20 | 4000 | 0 | -1 | -1 | -1 | 1 | -1 | -1 | 10.44 |
| | 20 | 100 | 0 | 2 | 4000 | 20 | 1 | -0.3 | -1 | -1 | -1 | 1 | 5.40 |
| | 20 | 75 | 0 | 10 | 8000 | 20 | 1 | -1 | -1 | -0.11 | -0.11 | 1 | 11.33 |
| | 0 | 75 | 20 | 20 | 13000 | 0 | -1 | -1 | 1 | 1 | 1 | -1 | 9.00 |
| | 0 | 100 | 0 | 10 | 13000 | 0 | -1 | -0.3 | -1 | -0.11 | 1 | -1 | 0.74 |
| | 20 | 75 | 0 | 20 | 13000 | 20 | 1 | -1 | -1 | 1 | 1 | 1 | 8.56 |
| | 20 | 75 | 20 | 10 | 4000 | 0 | 1 | -1 | 1 | -0.11 | -1 | -1 | 3.84 |
| | 0 | 150 | 0 | 20 | 8000 | 20 | -1 | 1 | -1 | 1 | -0.11 | 1 | 9.53 |
| | 20 | 100 | 20 | 10 | 8000 | 0 | 1 | -0.3 | 1 | -0.11 | -0.11 | -1 | 3.05 |
| | 0 | 150 | 0 | 10 | 13000 | 20 | -1 | 1 | -1 | -0.11 | 1 | 1 | 8.48 |
| | 20 | 150 | 20 | 20 | 13000 | 0 | 1 | 1 | 1 | 1 | 1 | -1 | 9.11 |
| | 0 | 150 | 20 | 2 | 8000 | 0 | -1 | 1 | 1 | -1 | -0.11 | -1 | 7.23 |
| | 0 | 150 | 20 | 10 | 4000 | 20 | -1 | 1 | 1 | -0.11 | -1 | 1 | 10.09 |
| | 20 | 100 | 20 | 20 | 4000 | 20 | 1 | -0.3 | 1 | 1 | -1 | 1 | 10.05 |
| | 20 | 75 | 20 | 2 | 13000 | 20 | 1 | -1 | 1 | -1 | 1 | 1 | 8.95 |
| | 20 | 150 | 20 | 2 | 13000 | 20 | 1 | 1 | 1 | -1 | 1 | 1 | 9.22 |
| V | 0 | 75 | 20 | 2 | 4000 | 20 | -1 | -1 | 1 | -1 | -1 | 1 | 8.44 |
| | 20 | 75 | 0 | 10 | 8000 | 20 | 1 | -1 | -1 | -0.11 | -0.11 | 1 | 9.48 |
| | 20 | 100 | 20 | 20 | 4000 | 20 | 1 | -0.3 | 1 | 1 | -1 | 1 | 9.53 |
| | 20 | 75 | 20 | 2 | 13000 | 20 | 1 | -1 | 1 | -1 | 1 | 1 | 9.05 |

**Table S2.** Results of model validation. EXP, experimental value; PRED, model prediction; lev, leverage; LLCI, CI95%, confidence interval at 95%; res, residuals.

| EXP | PRED | lev | $CI_{95\%}$ | res |
|---|---|---|---|---|
| 8.44 | 7.97 | 0.77 | 4.16 - 11.78 | -0.47 |
| 9.48 | 10.49 | 0.79 | 6.63 - 14.35 | 1.01 |
| 9.53 | 9.51 | 0.88 | 5.44 - 13.58 | -0.02 |
| 9.05 | 9.20 | 0.74 | 5.48 - 12.93 | 0.16 |

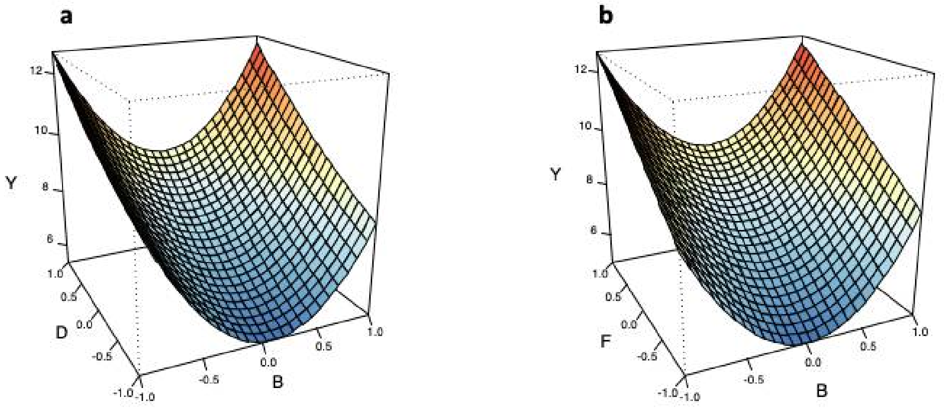


**Figure S2.** Response surfaces. a) Response (Y) predicted by the experimental design model as a function of D (centrifugation time) and B (volume of colloidal AgNPs solution), while keeping A = 0 (10 min), C = 0 (10 min), E = 0 (8000 rpm), and F= 1 (20 min); b). Response (Y) predicted by the experimental design model as a function of F (drying time after centrifugation) and B (volume of colloidal AgNPs solution), while keeping A = 0 (10 min), C = 0 (10 min), D= 1 (20 min), and E = 0 (8000 rpm).

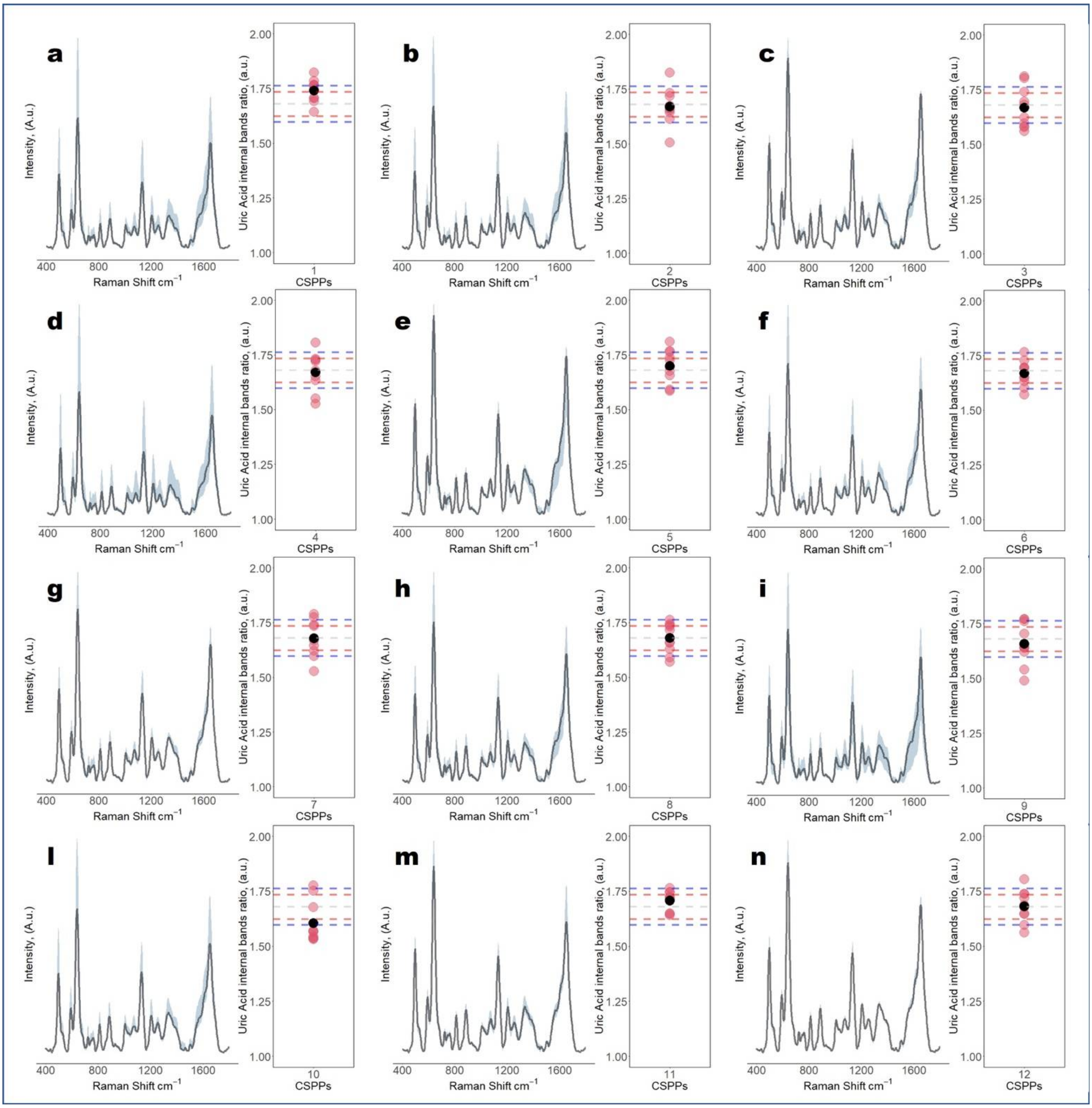


**Figure S3.** Reproducibility of the CSPP method (n=12). Black dots correspond to the mean value for the ratio between the 640 (skeletal ring deformation) and 1132 (C-N) cm$^{-1}$ bands of uric acid of 10 randomly chosen positions on the same CSPP. The red shaded lines span the interquartile range (IQR), calculated over the entire set of measurements, corresponding to the range covered by 50% of the data. The dashed blue lines mark the median and the extremes of the acceptance area (1.5 x IQR).

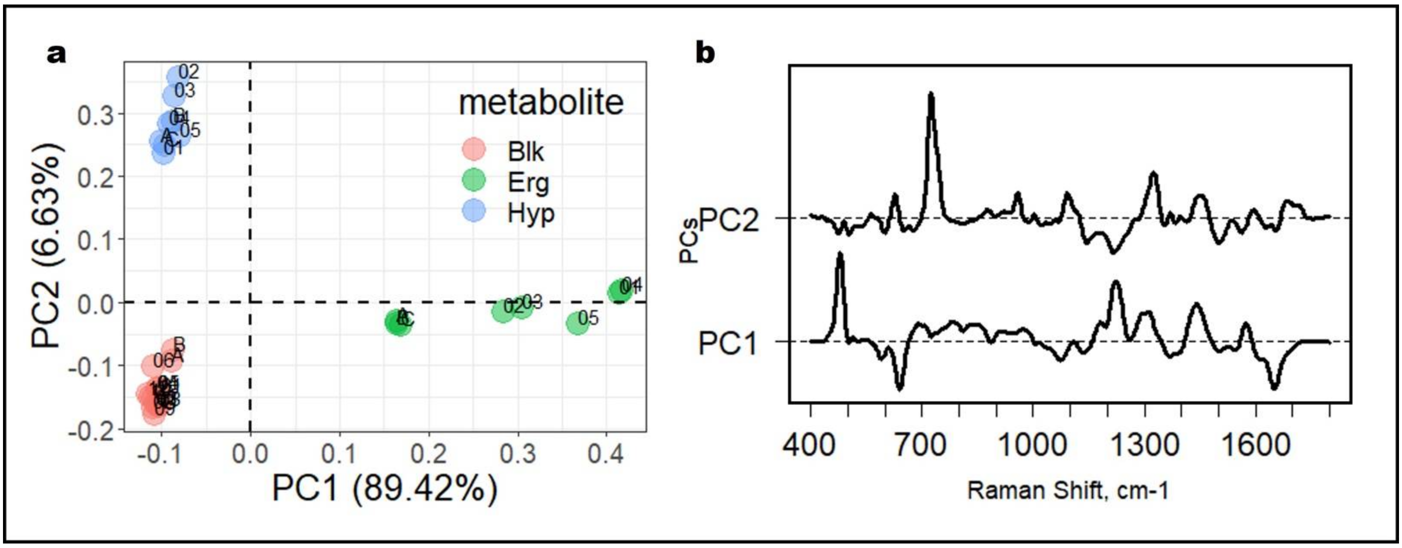


**Figure S4**. Principal components analysis. a) PC1-PC2 score plot of the dataset used in section. b) variable loadings profiles on PC1 and PC2. Blk, human serum; Erg, serum spiked with 25 µM ergothioneine; Hyp, human serum spiked with 50 µM hypoxanthine.
